# Culture-dependent and culture-independent characterization of potentially functional biphenyl-degrading bacterial community in response to extracellular organic matter from *Micrococcus luteus*

**DOI:** 10.1111/1751-7915.12266

**Published:** 2015-02-12

**Authors:** Xiao-Mei Su, Yin-Dong Liu, Muhammad Zaffar Hashmi, Lin-Xian Ding, Chao-Feng Shen

**Affiliations:** 1 Department of Environmental Engineering, College of Environmental and Resource Sciences, Zhejiang UniversityHangzhou, 310058, China; 2 College of Geography and Environmental Science, Zhejiang Normal UniversityJinhua, 321004, China

## Abstract

Biphenyl (BP)-degrading bacteria were identified to degrade various polychlorinated BP (PCB) congers in long-term PCB-contaminated sites. Exploring BP-degrading capability of potentially useful bacteria was performed for enhancing PCB bioremediation. In the present study, the bacterial composition of the PCB-contaminated sediment sample was first investigated. Then extracellular organic matter (EOM) from *Micrococcus luteus* was used to enhance BP biodegradation. The effect of the EOM on the composition of bacterial community was investigated by combining with culture-dependent and culture-independent methods. The obtained results indicate that *Proteobacteria* and *Actinobacteria* were predominant community in the PCB-contaminated sediment. EOM from *M. luteus* could stimulate the activity of some potentially difficult-to-culture BP degraders, which contribute to significant enhancement of BP biodegradation. The potentially difficult-to-culture bacteria in response to EOM addition were mainly *Rhodococcus* and *Pseudomonas* belonging to G*ammaproteobacteria* and *Actinobacteria* respectively. This study provides new insights into exploration of functional difficult-to-culture bacteria with EOM addition and points out broader BP/PCB degrading, which could be employed for enhancing PCB-bioremediation processes.

## Introduction

Polychlorinated biphenyls (PCBs) are toxic persistent pollutants that threaten both natural ecosystem and human health (Zanaroli *et al*., 2010). *In situ* PCB bioremediation have aroused increasing concern because they are less expensive and more environmentally sound than conventional methods (Leigh *et al*., 2006). A large number of bacteria have been identified and shown to cometabolize PCBs through the biphenyl (BP) catabolic pathway (Petrić *et al*., 2007). Indeed, research on aerobic PCB biodegradation bacteria isolated so far has mainly focused on BP-utilizing bacteria. Numerous phylogenetically diverse BP-utilizing bacteria that have the capability to transform several PCB congeners have been isolated (Abraham *et al*., 2002; Pieper, 2005). However, until recently, the full-scale bacterial remediation of PCB-contaminated environment performed not very well, because the activity and capability of BP/PCB-degrading strains soon decreased when exposed to natural environment. Although bioaugmentation of sites with degradative bacteria have been unsuccessful application in the field, efforts to stimulate indigenous BP/PCB-degrading bacteria have been promising (Leigh *et al*., 2006).

In the natural environment, it is very common for bacteria to survive under a wide variety of stress conditions by entering a ‘viable but non-culturable’ (VBNC) state, in which cells are intact and alive but fail to normally grow on the routine bacteriological media (Oliver, 2010). The high toxicity of PCBs and their low bioavailability exerted significant stress on indigenous microorganisms. It should be interesting to find out the survival and activity of bacteria exposed to such adverse conditions (Chávez *et al*., 2006). Although numerous BP/PCB-degrading bacteria have been isolated and studied, there are more phylogenetically diverse bacteria detected in the environment using molecular tools (Macedo *et al*., 2007). Undoubtedly, using conventional plate separation methods, only a small fraction of BP/PCB-degrading bacteria existed in nature can be obtained. Furthermore, it is common knowledge that artificial mixed cultures consisting of purified cultivable isolates from enrichment cultures are less efficient in BP/PCB degradation than mixed cultures (Mikesková *et al*., 2012; Uhlik *et al*., 2012). One reason for this discrepancy is that there are abundant VBNC or uncultured bacteria possessing BP/PCB-degrading abilities in mixed cultures (Tang *et al*., 2013). Hence, the resuscitation and stimulation of potential BP/PCB-degrading indigenous bacteria is crucial for *in situ* bioremediation of PCBs.

The most exciting development in reaction of VBNC bacteria is the role of extracellular protein secreted by *Micrococcus luteus*, known as a resuscitation-promoting factor (Rpf), which has been shown to promote the resuscitation and growth of high G + C Gram-positive organisms (Mukamolova *et al*., 1998; Su *et al*., 2013b). It is worth noting that these organisms contain well-known BP/PCB degraders, including *Rhodococcus*, *Arthrobacter*, *Bacillus* and *Microbacterium* (Pieper, 2005; Petrić *et al*., 2007). In addition, Ding and Yokota (2010) indicated that Rpf could also enhance the culturability of several other Gram negative. Hence, Rpf is capable of culturing difficult-to-culture bacteria involved in the BP/PCB degradation process and stimulating the activity of indigenous bacterial communities in PCB-contaminated environment. Mukamolova and colleagues (2006) indicated that Rpf control the culturability of several bacteria because of its muralytic activity. And at least two additional extracellular proteins in the *M. luteus* culture supernatant were also found to possess the same muralytic activity as Rpf protein. On the other hand, the recombinant Rpf protein presented lower activity than the native Rpf protein (purified from *M. luteus* culture supernatant), and both of them were prone to lose its activity after storage at 4°C for 1 week (Mukamolova *et al*., 2006). By analysing on the disadvantage of Rpf protein, extracellular organic matter (EOM) from *M. luteus* offers an attractive and cost-effective alternative additive for resuscitating and stimulating VBNC or difficult-to-culture bacteria, as well as enhancing the activity and degrading capability of indigenous bacteria in PCB-contaminated area.

The bacterial composition of the PCB-contaminated sediment sample was first investigated in the present study. Above all, to test the hypothesis that EOM from *M. luteus* was a feasible and effective additive for enhancing bacterial BP-degradation capability, the effect of EOM on BP biodegradation and bacterial community was assessed. Specifically, the degradation abilities, bacterial composition and abundance of enrichment cultures with different experimental treatment were investigated. In this work, we aimed to identify whether EOM could resuscitate the BP-degrading potential of VBNC or difficult-to-culture bacteria by isolating pure cultures unique to the enrichment culture with EOM addition. To our best knowledge, this is the first attempt to reveal the effect of EOM from *M. luteus* on BP-degrading bacterial communities using the combination of Illumina high-throughput sequencing and culture-dependent methodology.

## Results and discussion

### Bacterial composition in the PCB-contaminated sediment

**Fig 1:**
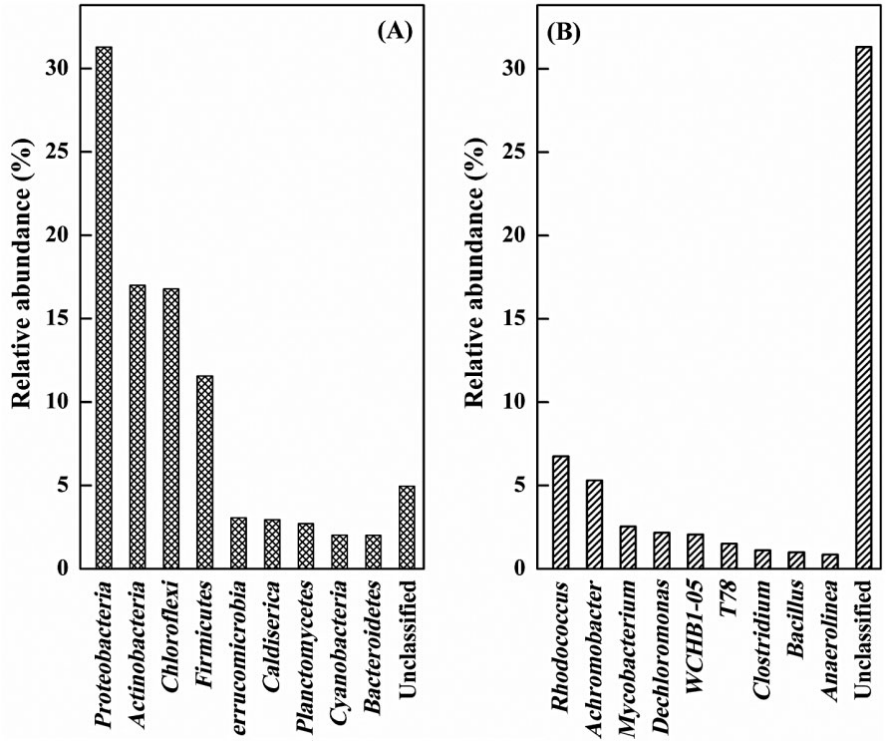
Taxonomic composition of bacterial community in the PCB-contaminated sediment sample. (A) Taxonomic affiliations at the phylum level. (B) Taxonomic affiliations at the genus level.

Illumina high-throughput sequencing was performed to determine the diversity and composition of the bacterial communities in the sediment sample. The top 10 dominant phyla/genera are shown in Fig. 1. As shown in Fig. 1A, of the 10 major phyla, the most predominant phylum was *Proteobacteria*, which represented 31.3% of the total bacteria. *Actinobacteria*, *Chloroflexi* and *Firmicutes* were the subdominant groups, constituting around 50% of the total population. At the genus level (Fig. 1B), the four most abundant genera, which belonged to *Rhodococcus*, *Achromobacter*, *Mycobacterium* and *Dechloromonas*, accounted for 18.8% of all sequences. The proportion of sequences assigned as unclassified constituted highly 31.3% of the total population. The composition of the bacterial community of the sediment sample identified in this study is in accordance with previous studies (Fukuda *et al*., 1998; Macedo *et al*., 2007; Uhlik *et al*., 2009), which are rather common abundant bacteria in PCB-contaminated environments adapted for biodegradation of the pollutants. To improve bioremediation practices, it is important to understand the composition of bacterial community in long-term PCB-contaminated sites (Petrić *et al*., 2011). These results indicate that genera *Rhodococcus* and *Achromobacter* with outstanding degradation abilities were widespread in long-term PCB-contaminated sediment, in which the total of 20 PCB congeners (from one to six chlorine atoms) concentration averaged 29.16, and the standard deviation was 0.72 μg g^−1^. In addition, it would be interesting to find that majority indigenous bacteria cannot be cultivated. Indeed, yet-to-be cultured bacteria probably account for the majority of the degradation activity among indigenous bacteria (Uhlik *et al*., 2012; Tang *et al*., 2013).

### Effect of EOM on BP degradation

**Fig 2:**
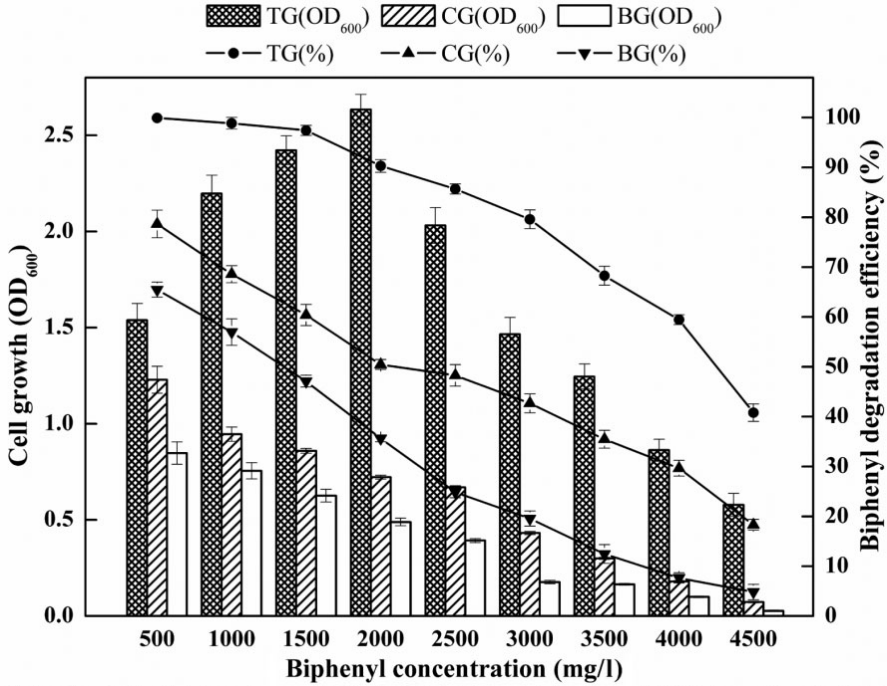
Comparison of tolerance concentration of biphenyl among different groups of enrichment cultures. TG: addition of EOM; CG: addition of autoclaved EOM; BG: without EOM. Error bars indicate the standard deviations of triplicate samples.

**Fig 3:**
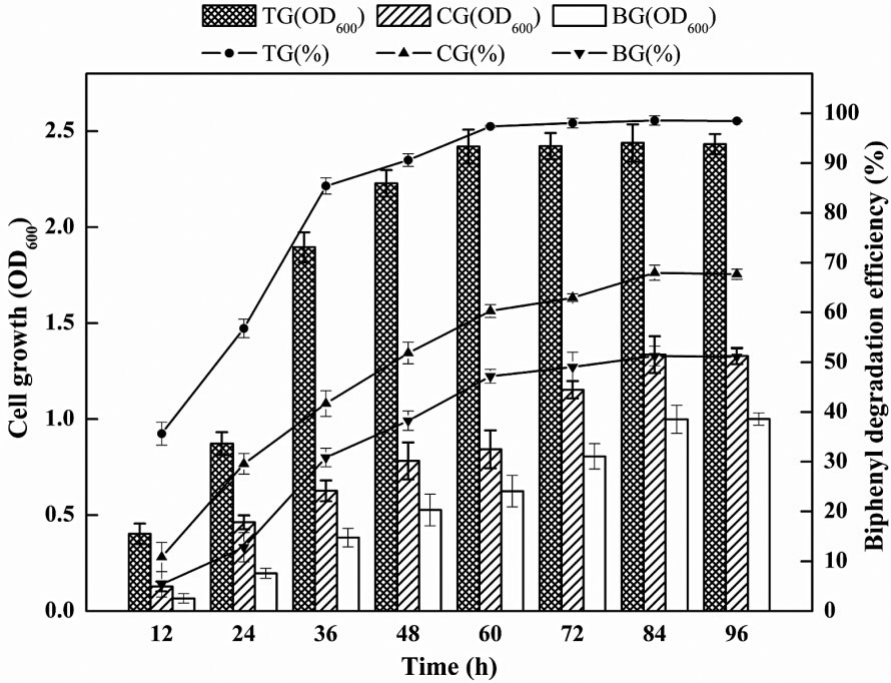
Comparison of biphenyl-degrading curve among different enrichment cultures at a concentration of 1500 mg l^−1^. TG: addition of EOM; CG: addition of autoclaved EOM; BG: without EOM. Error bars indicate the standard deviations of triplicate samples.

Based on the BP-degradation efficiency and cell growth (OD_600_), the effect of EOM on the BP tolerance concentration, BP-degrading curve and cell growth was investigated. The degradation of BP by enrichment samples under various BP concentrations (from 500 to 4500 mg l^−1^) is detailed in Fig. 2. As shown in Fig. 2, the BP-degradation efficiency of all the groups decreased with elevated BP concentrations. When BP concentration was increased from 500 to 3500 mg l^−1^, the BP-degradation efficiency decreased from 99.9% to 68.3% in treatment group (TG), whereas it decreased from 65.5% to 12.4% in blank group (BG). Under a BP concentration of 4000 mg l^−1^, BP-degradation efficiency reached 59.5% in TG, whereas the efficiency was 29.6% and 7.6% in CG and BG respectively. Similar to the trend of variations in BP-degradation efficiency, cell growth decreased with elevated BP concentrations in the CG and BG. However, the values of OD_600_ in TG underwent an increasing trend, reaching its maximum value of 2.63 at 2000 mg l^−1^, and then decreased to 0.58 at BP concentration of 4500 mg l^−1^. Meanwhile, under a BP concentration of 4000 mg l^−1^, the value of OD_600_ in TG was observed to be around fivefold and ninefold greater than that observed in control group (CG) and BG. Meanwhile, Fig. 3 depicts the changes in the BP-degradation curves and cell growth among the three groups. As shown in Fig. 3, the BP-degradation efficiency and cell growth were significantly enhanced in TG. After 60 h, the BP-degradation efficiency in TG reached up to 97.4%, whereas the efficiency was less than 60.3% in CG and 47.2% in BG. The overall trend in cell growth coincided with the degradation rate, and significant increases in the value of OD_600_ were recorded. These results indicated that TG with EOM addition maintained higher degradation efficiency and faster cell growth and was more tolerant to increasing BP concentration. Specifically, the BP-degradation efficiencies of TG were higher than those of CG, suggesting that EOM performed better than autoclaved EOM in enhancing BP degradation. The better performance of TG could be attributed to the positive effects of some proteins in EOM on cell growth and BP-degradation efficiency, because autoclaved EOM had the same constituents as EOM except proteins. In addition, the BP-degradation efficiencies of CG were higher than BG, suggesting that some polysaccharides of EOM may also play a role in enhancing BP degradation.

In previous studies, the resuscitation and stimulation function of proteins in the culture supernatants of *M. luteus* has been tentatively verified (Ding, 2004; Su *et al*., 2013b). This study further indicated that EOM from *M. luteus* as an efficient additive could significantly enhance the performance of BP biodegradation. Recent advances in PCB bioremediation processes have focused on selecting BP-degrading enrichment cultures to obtain BP/PCB-degrading community and identifying the key members involved in this process using culture-independent approaches (Cámara *et al*., 2004; Uhlik *et al*., 2012). Nevertheless, most of bacteria and enrichment cultures, which were obtained with high removal rates in the laboratory, showed exceptionally slow removal rates *in situ* (Abraham *et al*., 2002). Therefore, the challenge for PCB-bioremediation is to enhance the activities of bacterial communities involved in biodegradative processes and develop approaches to realize the full potential of BP/PCB degraders (Gomes *et al*., 2013). Clearly, culture-dependent studies can focus only on a limited number of bacteria that are not likely to represent the bacterial diversity in the environment, as there are abundant of VBNC or difficult-to-culture bacteria that are important players in bioremediation existed ubiquitously in PCB-contaminated (de Cárcer *et al*., 2007; Zanaroli *et al*., 2010). Although culture-independent molecular techniques can investigate the diversity of bacteria potentially responsible for ecologically relevant processes, to elucidate bacteria related function and genotype, it will be necessary to recover them and study their microbiology in pure cultures (Uhlik *et al*., 2012; Su *et al*., 2013b). Thus, EOM from *M. luteus* can be employed for culturing difficult-to-culture bacteria and for exploiting the potential environmental functions of VBNC or difficult-to-culture bacteria, which will demonstrate highly valuable in future bioremediation strategies.

### Effect of EOM on bacterial diversity and composition

Illumina high-throughput sequencing was adopted to investigate bacterial community diversity and richness among sediment sample and enrichment cultures (TG, CG and BG) (Table 1). In order to compare the bacterial species richness, operational taxonomic units (OTUs) were estimated by Chao1 estimator at a distance level of 3%. The total number of OTUs in TG (2272) with EOM addition was the largest among the enrichment cultures. And BG (1981) contained the least OTUs amount, indicating that TG with EOM addition exhibited the greatest bacterial richness. Furthermore, the bacterial phylotype richness can also be reflected using Shannon diversity index, which is generally used to demonstrate species diversity in a bacterial community, and it accounts for both evenness and abundance of the species present (Ma *et al*., 2013). Considering the fact that Shannon index of TG (5.60) was much higher than that of CG (5.02) and BG (4.87), it could be inferred that the enriched OTUs in TG community distributed more evenly than those in CG and BG. These results suggested that bacterial community diversity and richness increased with EOM addition.

**Table 1:** Alpha diversity of samples

`	OTUs	Chao 1 richness estimation	Shannon diversity
Sediment sample	5623	23 187	9.86
Enrichment sample TG	2272	10 561	5.60
Enrichment sample CG	2175	9803	5.02
Enrichment sample BG	1981	9259	4.87

**Fig 4:**
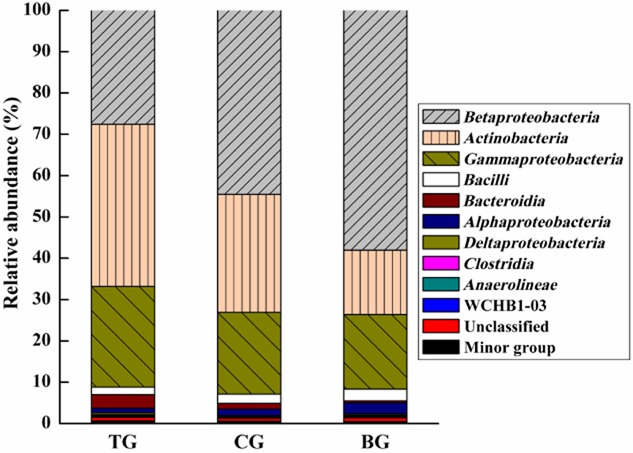
Taxonomic composition of bacterial communities at the class level in enrichment cultures. TG: addition of EOM; CG: addition of autoclaved EOM; BG: without EOM.

The effective bacterial sequences in the TG, CG and BG were all assigned to corresponding taxonomies. Figure 4 shows the taxonomic compositions at the class level for the three enrichment cultures, demonstrating a relative abundance greater than 0.1% in at least one enrichment culture. In total, 10 identified class were observed; *Bataproteobacteria*, *Actinobacteria* and *Gammaproteobacteria* were the dominant class, which were consistent with previous studies on bacterial community composition in polycyclic aromatic hydrocarbon and PCB exposed environments (Stach and Burns, 2002; Petrić *et al*., 2011). However, the relative abundance of each dominant class among the three enrichment cultures was distinct. Especially, the relative abundance of *Bataproteobacteria*, *Actinobacteria* and *Gammaproteobacteria* were 27.6%, 39.3% and 24.3% in TG, and 58.0%, 15.6% and 18.1% in BG. *Actinobacteria* were the most dominant bacterial community in TG instead of *Bataproteobacteria* in CG and BG. Thus, it could be inferred that after EOM addition, *Actinobacteria* and *Gammaproteobacteria* were greatly enriched. Moreover, a small fraction of class *Bacteroidia* accounting for less than 0.1% of total community showed a similar trend, which the order of abundance was TG > CG > BG. The *Bacteroidia* class is believed to be involved in the degradation of aromatic compounds (Xu *et al*., 2012). Petrić and colleagues (2011) had previously reported that *Actinobacteria* and *Bacteroides* were the predominant phyla in bioremediation of PCB-contaminated soil. In view of these results, it could be concluded that EOM from *M. luteus* greatly affects the composition and abundance of bacterial communities closely related to BP/PCB degradation.

**Fig 5:**
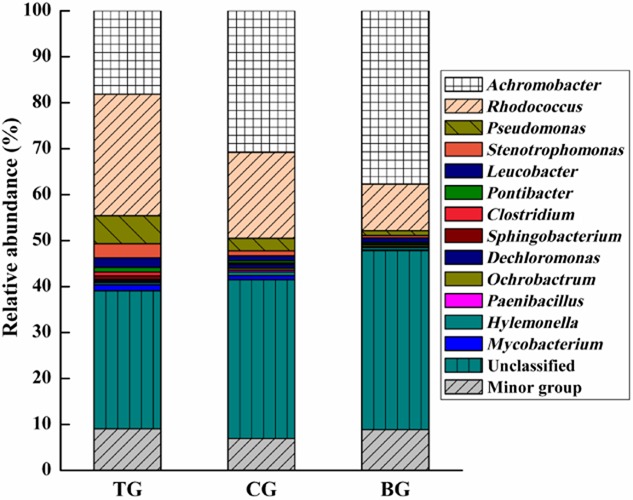
Taxonomic composition of bacterial communities at the genus level in enrichment cultures. TG: addition of EOM; CG: addition of autoclaved EOM; BG: without EOM.

To further explore the variation in bacterial community with EOM addition, bacterial abundance was also analysed more specifically at the genus level. As Fig. 5 illustrates, a relative abundance greater than 0.1% in at least one enrichment culture is summarized. Other genera were grouped into minor groups. At the genus level, the majority of sequences were affiliated to five types, such as *Achromobacter*, *Rhodococcus*, *Pseudomonas*, *Stenotrophomonas* and unclassified bacteria. And the composition of TG with EOM addition was also reflected to be dramatically different. The genus *Rhodococcus* dominated TG (26.4% of total reads) community, followed by *Achromobacter* (18.2%), *Pseudomonas* (6.1%) and *Stenotrophomonas* (3.1%). However, the genus *Achromobacter* was the most dominant bacterial community, accounting for 30.8% and 37.69% of the total reads in CG and BG community respectively. Notably, the order of abundance for the genera *Rhodococcus*, *Pseudomonas* and *Stenotrophomonas* was TG > CG > BG. The discrepancy between TG and CG could be attributed to the function of some proteins in EOM. Compared with BG, CG with autoclaved EOM had higher relative abundance of the three genera which may be attributable to the stimulation function of polysaccharides in EOM. Overall, well-known PCB degraders (*Rhodococcus*, *Pseudomonas* and *Stenotrophomonas*) were greatly abundant after EOM addition (Macedo *et al*., 2007; Correa *et al*., 2010). The results in this study is consistent with earlier reports that Rpf could promote the resuscitation and growth of not only high G + C Gram-positive organisms, but also several other Gram-negative organisms (Mukamolova *et al*., 2006; Oliver, 2010; Su *et al*., 2013a).

It was interesting to observe that a large number of unclassified sequences (30.1–38.9% of total reads) were found in the enrichment cultures, suggesting that a wide variety of novel species or yet-to-be cultured bacteria may inhabit complex BP enrichment cultures communities. The results indicated that some uncultured or VBNC bacteria frequently detected in PCB-contaminated sites and BP enrichment cultures may be highly correlated to the functions and performances of BP/PCB biodegradation process. This is in agreement with previous investigation on degradative bacterial communities in BP/PCB-contaminated environments (Abraham *et al*., 2002; Uhlik *et al*., 2012). Indeed, the traditionally isolated genera of bacteria possessing the capability for pollutants degradation represent only a small fraction of the total diversity in nature. In this study, bacteria in the enrichment culture were exposed to high concentrations of BP and progressive nutrient depletion, both of which may initiate the VBNC state. Tang and colleagues (2013) found that many clones related to BP/PCB degradation were uncultured bacteria. Furthermore, *Actinobacteria* as the predominance of the phylum in the BP/PCB-degrading community process was prone to enter VBNC state in which they have significantly reduced metabolic activity and lose culturability (Keep *et al*., 2006; Mukamolova *et al*., 2006). In addition, Mukamolova and colleagues (2006) had previously shown that the culturability of several *Actinobacteria* is controlled by Rpf. And Rpf homologues are widespread throughout the *Actinobacteria*. Meanwhile, Schroeckh and Martin (2006) found that Rpf could resuscitate *Actinobacteria* and was a useful tool for isolating new actinobacterial species. Typically, *Rhodococcus* is known to enter into a VBNC state quite rapidly under adverse conditions, such as *R. rhodochrous* and *R. fascians* (Shleeva *et al*., 2002). The results supported the hypothesis that the positive effects of EOM on BP-degrading capability enhancement was mainly caused by its ability to resuscitate and stimulate VBNC bacteria, especially for BP/PCB-degrader *Rhodococcus*-like populations. Therefore, it may be inferred that EOM from *M*. *luteus* containing the Rpf and Rpf-like proteins provides a useful approach for isolating novel species or yet-to-be cultured bacteria and realizing the full potential of BP/PCB degraders.

### Isolation and phylogenetic analysis of bacteria

**Fig 6:**
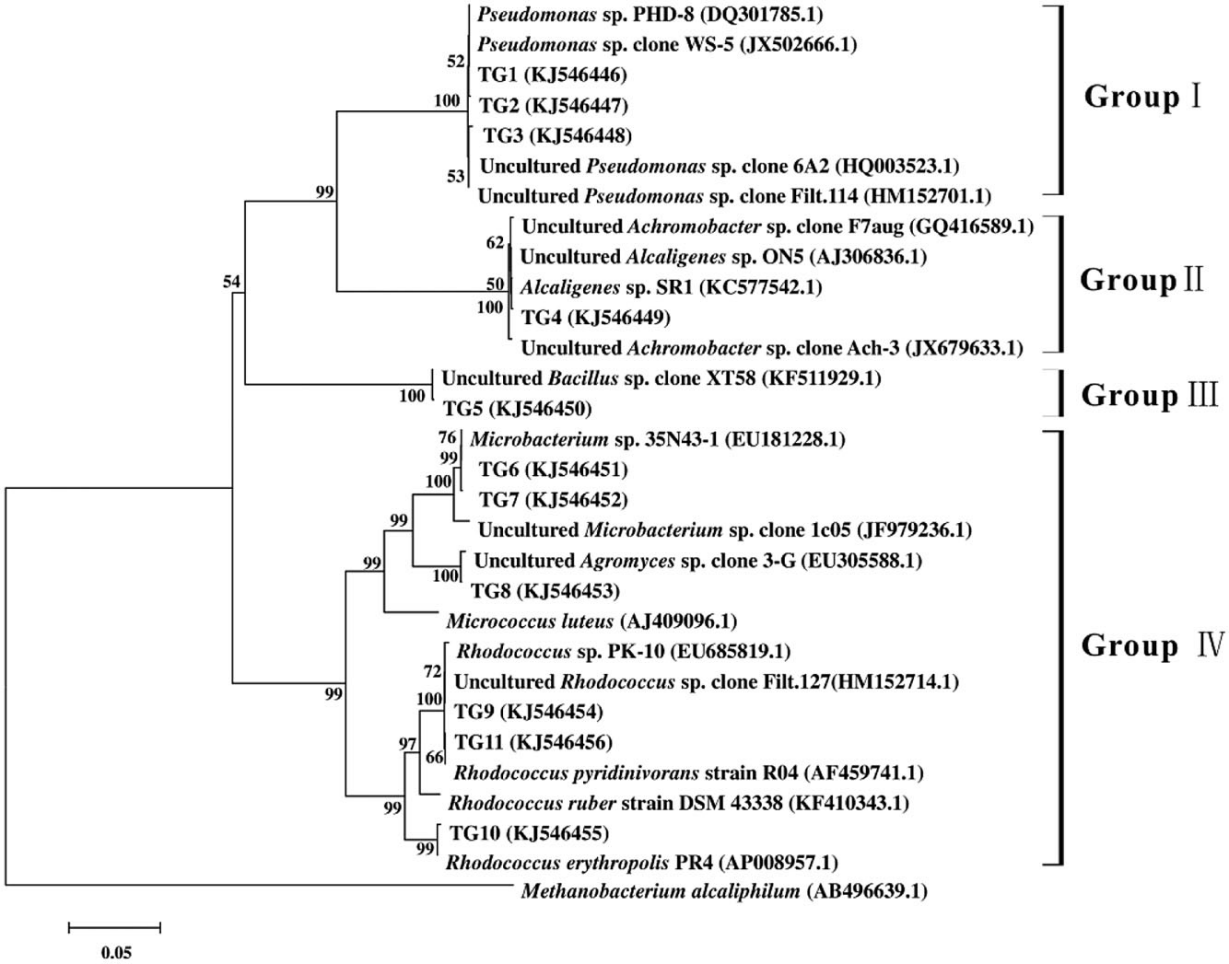
Phylogenetic tree of bacterial 16S rRNA gene sequences, including eleven isolates unique to enrichment culture with EOM addition and 18 of their most similar GenBank sequences. Bootstrap values (> 50%) are showed at branch points. The bar represents a sequence distance of 0.1.

In order to further verify the resuscitating function of EOM, the three enrichment cultures (TG, CG and BG) were also investigated using a culture-dependent method. Colonies that were unique in TG, with no counterpart in CG and BG, were isolated using a modified most probable number method. Eleven unique strains in TG (TG1–TG11) were isolated on mineral salts agar plates. Phylogenetic analysis was based on 16S rDNA sequences. Closely matching representatives were determined by a Blast search at GenBank. Figure 6 presents an overview of the 11 unique strains based phylogenetic tree, generated by including representative members. Overall, at the class level, strains belonging exclusively to TG can be broadly divided into four subgroups. The most dominant classified subgroup was *Actinobacteria* (subgroups IV), accounting for 54.5% (6/11) of the total strains. G*ammaproteobacteria* (subgroups I) was the subdominant group, which constituted 27.2% of the total strains. *Bataproteobacteria* (subgroups II) and *Bacilli* (subgroups III) only contributed to about 18% of the total population. The results are consistent with the investigations on variation of community composition in TG analysed by Illumina high-throughput sequencing. And the analyses based on the culture-dependent method further confirmed that EOM could resuscitate functional bacteria for BP degradation, especially the *Actinobacteria*.

Moreover, it was worth noting that strains TG6–TG11 belonging to genera *Microbacterium*, *Agromyce*s and *Rhodococcus* are highly homologous with *M. luteus*. Furthermore, it is evident that strain TG9 represents a novel species of the genus *Rhodococcus*, for which the name *Rhodococcus biphenylivorans* sp. nov. is proposed (Su *et al*., 2015). In addition, VBNC state of the strain TG9^T^ in response to low temperature and oligotrophic nutrients was verified (data not shown). This is agreement with previous reports that Rpf could promote the resuscitation and growth of Gram-positive organisms (Keep *et al*., 2006; Su *et al*., 2013a). By contrast, strains TG1–TG4 belonging to genera *Pseudnomonas* and *Achromobacter* are Gram-negative bacteria, which were well supported by the observation that Rpf also resuscitated the growth of several other Gram-negative bacteria (Ding and Yokota, 2010; Su *et al*., 2013b). Additionally, these bacteria unique to TG were closely related to uncultured bacteria, which are known for their catabolic diversity in degradation of aromatic compounds (Zanaroli *et al*., 2010; Xu *et al*., 2012). In view of these results, it could be inferred that after EOM addition, functional difficult-to-culture bacteria belonging to genera *Microbacterium*, *Agromyce*s, *Rhodococcus*, *Pseudnomonas* and *Achromobacter* recovered their culturability.

### Application prospect of EOM from *M. luteus*

In the present study, the influence of EOM from *M. luteus* on the BP-degradation capability and composition of bacterial community in PCB-contaminated sediment was first evaluated by combining with culture-dependent and culture-independent methods. The obtained results provided evidence that EOM from *M. luteus* could significantly enhance the BP-degrading capability, which could be attributed to enrichment of some potentially VBNC or difficult-to-culture BP/PCB degraders. In addition, it was evident that the mainly functional bacteria in response to EOM addition were *Actinobacteria*, which were prone to enter into VBNC state and showed a low degrading activity in long-term PCB-contaminated sediment.

Recent advances in the bioremediation of PCB-contaminated sites have focused on the development of ways to stimulate the activities of indigenous BP/PCB-degrading community (Leigh *et al*., 2006; Gomes *et al*., 2013). However, the application of PCB bioremediation is still inefficient and not well established up to now. It has been known that diverse BP-utilizing bacteria have the capability to transform several PCB congeners by *bph* encoded BP pathway (Cámara *et al*., 2004). And numerous studies have focused on the BP-degrading bacterial community and BP degradation pathway in order to explore a competitive advantage of PCB degraders and establish optimized PCB-bioremediation processes (Pieper, 2005; Uhlik *et al*., 2009). However, highly efficient BP/PCB-degrading community in the laboratory experiments showed lower efficiency and survived poorly when these cultures were inoculated in PCB-contaminated sites. Thus, it will be of critical importance to stimulate functional bacteria to enhance their BP/PCB degrading capabilities for *in situ* bioremediation. The present study demonstrated that EOM from *M. luteus* is an efficient additive which can significantly enhance BP biodegradation by recovering and stimulating the potentially functional BP-degrading community. It provided new insight into the exploration of potentially functional bacteria for enhancing in situ bioremediation, which could be inferred that the addition of EOM to PCB-contaminated areas holds great potential for the efficient and cost-effective bioremediation of PCB-contaminated environments.

### Conclusions

The obtained results suggest that *Proteobacteria* and *Actinobacteria* were two predominant classes in long-term PCB-contaminated sediment. EOM from *M. luteus* enhanced the performance of BP biodegradation, which could be attributed to stimulation of the growth and activity of some potentially BP/PCB-degraders. Illumina high-throughput sequencing and culture-dependent methods indicated that the genera of *Rhodococcus* and *Pseudomonas* which were related to BP/PCB-degradation were greatly abundant after EOM addition, and potentially difficult-to-culture bacteria in response to EOM addition were mainly *Actinobacteria*. This study provides new insights into the identity of as-yet uncultured and unclassified bacteria actively metabolizing BP/PCB with EOM addition, and points out broader BP/PCB-degrading community which could be employed in bioremediation of sites.

## Experimental procedures

### Preparation of EOM from *M. luteus*

*Micrococcus luteus* IAM 14879 (= NCIMB 13267) used in this study had previously been described (Mukamolova *et al*., 1998; Ding, 2004). The pure culture was inoculated at 30°C on a rotary shaker at 160 r.p.m. for 36 h in modified lactate minimal medium (LMM) of Kaprelyants and Kell (1992). Then, the pre-culture was grown under the same culture conditions until the cells reached the stationary phase. The obtained fermentation broth was centrifuged (8000 r.p.m., 15 min) to separate the cells and then filtered through a 0.22 μm filter to remove floating cells. Finally, EOM was obtained and stored at −20°C for further experiments. The pH and redox potential (Eh) of EOM were 8.8 and −106 mV respectively. The concentration of total carbon, nitrogen, phosphorus and sulphur of EOM were 867.8, 279.3, 99.8 and 42.1 mg l^−1^ respectively. The main components of EOM were polysaccharides and proteins, and their concentrations were 405.7 and 25.1 mg l^−1^ respectively. Especially, Rpf protein was dominated in the proteins of EOM (Su *et al*., 2015).

### Enrichment and cultivation

A PCB-contaminated sediment sample was obtained from a river very close to the e-waste recycling site in Luqiao Town of Taizhou City, which has been involved in e-waste disassembly for nearly 30 years (Shen *et al*., 2008; Chen *et al*., 2010; 2012). The sediment (4%, w/v) was incubated in a mineral salts medium (Su *et al*., 2013b), which BP (reagent grade, Sigma-Aldrich) was added as the sole carbon and energy source at an initial concentration of 500 mg l^−1^. Cultures were cultivated in conical flasks at 30°C on a rotary shaker at 180 r.p.m. Initial enrichments were transferred into fresh medium with 5% (v/v) inoculum after 5 days of cultivation. The BP concentration was increased in steps of 500 mg l^−1^ until a final concentration of 2000 mg l^−1^ was reached. Throughout the entire enrichment period, an equal amount EOM (10%, v/v), autoclaved EOM (sterilized at 121°C for 15 min) and LMM were added to TG, CG and BG respectively. Finally, the obtained enrichment samples were subjected to three different treatments, including TG, CG and BG.

### BP degradation of enrichment cultures

The BP degradation capability of the enrichment samples (TG, CG and BG) were assessed by investigating the BP-tolerance concentrations and BP-degradation curve of the samples described elsewhere (Su *et al*., 2013b). Briefly, after cultivating for 64 h at 30°C on a rotary shaker, BP-tolerance concentrations were investigated with the concentration of BP varying from 500 to 4500 mg l^−1^ in steps of 500 mg l^−1^. And the BP-degradation and cell growth curve was depicted at 12 h intervals within 96 h under a BP concentration of 1500 mg l^−1^. Lastly, the cell growth (OD_600_) and BP-degradation efficiency of each experimental culture were measured as previously described (Su *et al*., 2013b). All of the experiments were performed in triplicate, and standard deviation (SD) was calculated by SPSS software (version 18.0, Chicago, IL, USA) for analysis of statistical significance of the triplicate samples.

### DNA extraction

After lyophilization, DNA extraction of sediment sample was carried out using a beating method (FastDNATM SPIN Kit for Soil, Bio101, USA) following the manufacturer's protocol. For the enrichment samples and pure bacterial cultures, DNA extraction was performed using the EZ-10 spin column genomic DNA miniprep kit (Bio Basic, Canada) according to the manufacturer's instructions. The DNA extracts were stored at −20°C for further analysis.

### Illumina high-throughput sequencing

Illumina high-throughput sequencing was performed to determine the diversity and composition of the bacterial communities in the sediment and enrichment samples (Capodicasa *et al*., 2009). PCR amplifications were conducted with the 515F/806R primer set that amplifies the V4 region of the 16S rRNA gene. The reverse primer contained a 6 bp error-correcting barcode unique to each sample. DNA was amplified following the protocol described by (Magoč and Salzberg, 2011). Sequencing was conducted on an Illumina MiSeq platform. Sequences were analysed with the Quantitative Insights Into Microbial Ecology software package and UPARSE pipeline (Wang *et al*., 2007), and were assigned to OTUs at 97% similarity. All sequences have been deposited in GenBank short-read archive (SRA: SRS632100).

### Isolation and phylogenetic analysis of bacteria

The enrichment cultures TG, CG and BG were diluted respectively in 10-fold steps. Then serial dilutions (from 10^2^-fold to 10^8^-fold) was plated on mineral-salts agar plates with BP as the carbon source. Colonies that were unique in the TG, with no counterpart in the CG and BG, were selected. All isolates were purified and stored at 4°C for further identification. Genomic DNA was extracted from the pure bacterial cultures and then was amplified by PCR using primers 8F/1541R as described previously (Su *et al*., 2013b). Phylograms were constructed by the neighbour-joining method with 1000 replicate trees in the MEGA6 computer software program (Tamura *et al*., 2013). All sequences reported in this study had been submitted to NCBI GenBank under accession numbers KJ546446-KJ546456.

## Conflict and interest

None declared.
